# Diagnostic Performance and Accuracy of the MNA-SF against GLIM Criteria in Community-Dwelling Older Adults from Poland

**DOI:** 10.3390/nu13072183

**Published:** 2021-06-24

**Authors:** Aleksandra Kaluźniak-Szymanowska, Roma Krzymińska-Siemaszko, Marta Lewandowicz, Ewa Deskur-Śmielecka, Katarzyna Stachnik, Katarzyna Wieczorowska-Tobis

**Affiliations:** Department of Palliative Medicine, Poznan University of Medical Sciences, 61-245 Poznan, Poland; krzyminskasiemaszko@ump.edu.pl (R.K.-S.); dietetyk.martalewandowicz@gmail.com (M.L.); edeskur@ump.edu.pl (E.D.-Ś.); kstachnik@ump.edu.pl (K.S.); kwt@tobis.pl (K.W.-T.)

**Keywords:** malnutrition, diagnosis, older adults

## Abstract

Up to 28% of elderly residents in Europe are at risk of malnutrition. As uniform diagnostic criteria for malnutrition have not been formulated, in autumn 2018, the Global Leadership Initiative on Malnutrition (GLIM) presented a consensus on its diagnosis. According to the consensus, the diagnosis of malnutrition requires a positive screening test result for the risk of malnutrition, and the presence of at least one etiologic and one phenotypic criterion. This study aimed to assess the diagnostic performance and accuracy of the Mini Nutritional Assessment—Short Form (MNA-SF) against GLIM criteria. The analysis involved 273 community-dwelling volunteers aged ≥ 60 years. All participants were screened for malnutrition with the MNA-SF questionnaire. Next, the GLIM phenotypic and etiologic criteria were assessed in all subjects. Based on the presence of at least one phenotypic and one etiologic criterion, malnutrition was diagnosed in more than one-third of participants (*n* = 103, 37.7%). According to the MNA-SF, only 7.3% of subjects had malnutrition, and 28.2% were at risk of malnutrition. The agreement between the MNA-SF score and the GLIM criteria were observed in only 22.3% of the population. The sensitivity and specificity of MNA-SF against the GLIM criteria were fair (59.2% and 78.8%, respectively). The area under the curve (AUC) was 0.77, indicating the fair ability of MNA-SF to diagnose malnutrition. Based on the present study results, the best solution may be an optional replacement of the screening tool in the first step of the GLIM algorithm with clinical suspicion of malnutrition.

## 1. Introduction

The global number of older adults with malnutrition is constantly increasing. The main causes of this phenomenon are demographic changes, the increasing proportion of elderly subjects in society, and the higher risk of poor nutritional status in elderly people compared to younger subjects [1,2,3]. The most common reason for malnutrition is an insufficient intake of calories (concerning requirements) [4,5,6]. Malnutrition may also accompany inflammatory diseases associated with increased basal metabolic rate and changes in body composition [7,8].

Older people are at particular risk of malnutrition due to age-related physiologic changes, multimorbidity, psychological and socio-economic problems [9,10,11]. Malnutrition in elderly subjects is associated with increased risk of falls, disability, overall morbidity and mortality, health-related costs, and decreased quality of life [2,12,13,14]. The systematic review and meta-analysis performed by Leij-Halfwerket al. [14] demonstrated that up to 28% of older adults in Europe are at risk of malnutrition, which was assessed with various diagnostic tools.

As uniform diagnostic criteria for malnutrition have not been formulated, in 2015, the European Society for Clinical Nutrition and Metabolism (ESPEN) introduced two proposals on how to diagnose malnutrition. The first one was body mass index (BMI) < 18.5 kg/m^2^ and the other was unintentional body mass loss >10% in any time or >5% in 3 months, associated with a low BMI (defined as BMI < 20 kg/m^2^ in subjects <70 years, and BMI < 22 kg/m^2^ in subjects ≥70 years) or low muscle mass (defined as fat-free mass index (FFMI) < 15 kg/m^2^ in women and <17 kg/m^2^ in men) [15]. These criteria were based on phenotype features of malnutrition, but they did not consider the causes. To fill this gap, in 2018, the Global Leadership Initiative on Malnutrition (GLIM), formed by experts from the American Society of Parenteral and Enteral Nutrition (ASPEN), ESPEN, the Latin American Federation of Parenteral and Enteral Nutrition (FELANPE), and the Parenteral and Enteral Nutrition Society of Asia (PENSA), presented a consensus combining phenotypic and etiologic features of malnutrition in a two-step diagnostic process [7]. According to the consensus, malnutrition can be diagnosed if the results of a screening test indicate the risk of malnutrition, and a subject has at least one etiologic criterion (presence of a disease and/or inflammatory state, or limited intake/absorption of food) and at least one phenotype criterion (unintentional body mass loss, or low BMI, or low muscle mass) [7].

The first step of the diagnostic procedure consists of a screening test with a validated questionnaire, such as Nutritional Risk Screening-2002 (NRS-2002), Mini Nutritional Assessment—Short Form (MNA-SF), Malnutrition Universal Screening Tool (MUST), or Subjective Global Assessment (SGA). In elderly subjects, the MNA-SF is most frequently used to assess the risk of malnutrition [16,17,18,19]. The questionnaire comprises objective features of malnutrition, such as decreased food intake, body mass loss, and the presence of an acute illness or stress during the preceding three months. Additionally, it assesses the subject’s mobility and limitations, the presence of any neuropsychological disorders (depression or dementia), and body mass index. There is vast literature demonstrating the diagnostic performance of the NRS-2002, MUST, and SGA as malnutrition diagnostic tools against the GLIM criteria [20,21,22]. In contrast, the diagnostic performance of the MNA-SF in elderly subjects has not been extensively studied [23,24].

This study aimed to evaluate the diagnostic performance and accuracy of the MNA-SF against phenotypic and etiologic GLIM criteria and to emphasize the importance of clinical suspicion in the diagnostics of malnutrition.

## 2. Materials and Methods

We performed a cross-sectional study involving 273 community-dwelling volunteers ≥60 years of age (60–98 years) living in Poznan, Poland. Women (*n* = 178) accounted for 65.2% of the total study population. Each subject provided written informed consent prior to the study conducted under the Declaration of Helsinki. The study protocol was approved by the Bioethical Committee of the Poznan University Medical Sciences, Poland (approval No. 888/19).

### 2.1. Inclusion and Exlusion Criteria

The inclusion criteria were cognitive efficiency (defined as Abbreviated Mental Test Score (AMTS) ≥ 7) and the ability to maintain a standing position (necessary for body height measurement and body composition analysis). Subjects were excluded from the study if they had an artificial cardiac pacemaker, metal implants, or peripheral edemas—conditions that preclude body composition assessment with the bioimpedance method (BIA). Other exclusion criteria were impairment in oral intake and active cancer.

### 2.2. Study Protocol

In all participants, we performed a screening for malnutrition risk with the MNA-SF. Malnutrition was subsequently assessed with the GLIM criteria.

#### 2.2.1. GLIM Diagnostic Criteria for Malnutrition

In the present study, we diagnosed malnutrition based on the GLIM criteria in subjects with at least one etiologic criterion and at least one phenotypic criterion, regardless of the MNA-SF score.

Etiologic criteria:

(1) Reduced food intake was acknowledged in persons who declared at least moderate decrease in the number of meals or amount of food in the past three months;
(2) Disease burden/inflammatory condition was acknowledged if at least one chronic disease associated with chronic or periodic inflammation (e.g., chronic obstructive pulmonary disease, chronic heart failure, or chronic kidney disease) or elevated C-reactive protein levels (>10 mg/L) were identified in the subjects’ medical records.

Phenotypic criteria:

(1) Weight loss was acknowledged in persons who declared unintentional weight loss of at least 1 kg in the past three months;
(2) Low body mass index: <20 kg/m^2^ in subjects at age <70, and <22 kg/m^2^ in participants ≥70 years of age;
(3) Low muscle mass (LMM). Muscle mass was assessed based on the appendicular lean mass (ALM) index (i.e., the sum of the lean mass of the upper and lower limbs (kg) divided by the squared height (m^2^)) derived with the BIA method (InBody 120 analyzer, Biospace, Seoul, Korea). The ALM index below cut-off points for the Polish population (5.6 kg/m^2^ in women and 7.4 kg/m^2^ in men) were acknowledged low muscle mass [25].

The InBody 120 analyzer (assesses segmental impedance (right arm, left arm, trunk, right leg, left leg). The parameters used for further analysis were weight, BMI, skeletal muscle mass, segmental lean mass, fat mass, and percentage of fat mass.

According to the GLIM criteria, the severity of malnutrition was graded based on phenotypic criteria [7].

#### 2.2.2. MNA-SF Questionnaire

The Mini Nutritional Assessment—Short Form is one of the questionnaires recommended as a screening tool for malnutrition risk by the GLIM experts. Its psychometric properties were assessed in the present study.

The MNA-SF contains six questions regarding (1) decrease in food intake, (2) weight loss, (3) mobility, (4) psychological distress or acute disease, (5) neuropsychological problems, and (6) body mass index. The maximum score is 14. A score < 12 indicates a risk of malnutrition, and <7 indicates malnutrition. The cut-off point of 11 was used in the analysis of the MNA-SF psychometric properties against the GLIM criteria.

### 2.3. Statistical Analysis

Quantitative data are shown as means and standard deviations (SDs), and qualitative variables as numbers (*n*) and percentages (%). The normality of all quantitative variables were verified with the Shapiro–Wilk test. The equality of the variances was checked with Levene’s test. We used the following tests to assess differences between the groups, Student’s *t*-test for variables with normal distribution and equal variances, Cochrane–Cox test for variables with normal distribution and lack of homogeneity of variance, and Mann–Whitney U test for data with non-normal distribution. Qualitative variables were compared between groups with a chi-square test with the Yates correction for continuity.

To evaluate the MNA-SF diagnostic performance against the GLIM criteria, the following parameters were calculated: sensitivity, specificity, positive predictive value (PPV), negative predictive value (NPV), as well as the area under the ROC curve (AUC) and the kappa coefficient. Sensitivity reflects the percentage of subjects with malnutrition (based on the GLIM diagnostics criteria) who had a positive result on a screening test (an MNA-SF score indicating malnutrition or risk of malnutrition). Specificity refers to the percentage of subjects without malnutrition (based on the GLIM criteria) who had negative screening test results (an MNA-SF score indicating good nutritional status). Sensitivity and specificity were classified as good (>80%), fair (50–80%), or poor (<50%) [26]. The PPV reflects the probability that a person with a positive MNA-SF result is malnourished based on the GLIM criteria. The NPV measures the probability that a subject with a negative result of an MNA-SF does not have malnutrition based on the GLIM criteria. The AUC is a measure of the overall diagnostic accuracy of a test. An AUC > 0.8 indicates good, 0.6–0.8 fair, and <0.6 indicates poor diagnostics accuracy. The agreement between the MNA-SF score and the GLIM criteria were considered very good if the kappa coefficient was above 0.8, good if the kappa coefficient was 0.61–0.8, moderate if the kappa coefficient was 0.41–0.6, fair if the kappa coefficient was 0.21–0.4, and poor if the kappa coefficient was below 0.2 [26].

A *p-*value below 0.05 was considered significant. The statistical analysis was performed with STATISTICA 12.0 software (StatSoft, Cracow, Poland).

## 3. Results

### 3.1. Characteristics of the Study Group

The study population consisted of 287 subjects aged ≥60 years (mean age of 72.1 ± 7.7 years). On average, the subjects had three chronic diseases. One-third of the study population was living alone (33.3%), and one out of seven participants was a rural resident (15.4%).

Table 1 shows the characteristics of the total study population, subjects with malnutrition based on the GLIM criteria, and persons without malnutrition. Subjects with malnutrition based on the GLIM criteria tended to be older (a *p* of borderline significance) and had lower MNA-SF scores (*p* < 0.0001). They had lower body mass (*p* < 0.0001), a lower BMI (*p* < 0.0001), and lower muscle mass as assessed with the ALM index (*p* < 0.0001). They also had lower body fat mass (*p* < 0.0001), lower muscle mass (*p* < 0.0001), and lower fat-free mass (*p* < 0.0001) compared to subjects without malnutrition.

### 3.2. Prevalence of Malnutrition

**Figure 1:**
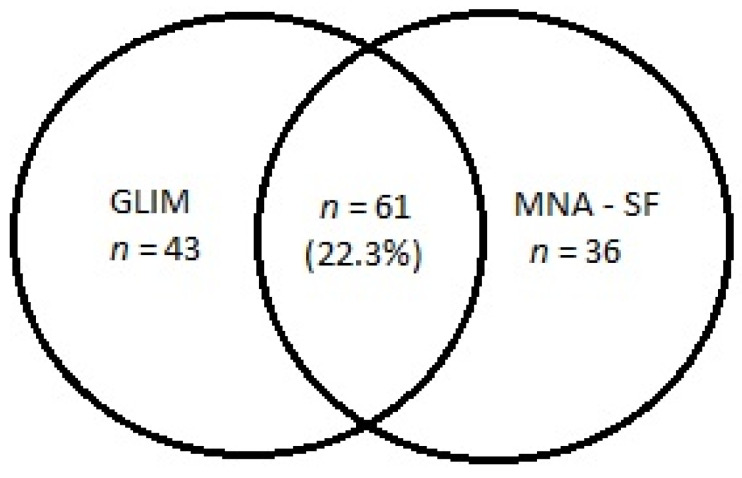
The number of subjects with malnutrition diagnosed based on the GLIM criteria and the MNA-SF score (malnutrition or risk of malnutrition). Notes: GLIM, Global Leadership Initiative on Malnutrition; MNA-SF, Mini Nutritional Assessment-Short Form.

Table 2 shows the prevalence of etiologic and phenotypic GLIM criteria and impaired nutritional status based on the MNA-SF score. Almost all participants (98.2%) had at least one etiologic criterion, and 38.1% of subjects had at least one phenotypic criterion. More than one-third of the population (37.3%) had malnutrition, based on the simultaneous presence of at least one phenotypic and one etiologic GLIM criterion. Based on the MNA-SF score, only 7.3% of participants had malnutrition, and 28.2% of subjects were at risk of malnutrition. The agreement between the MNA-SF and the GLIM criteria were as low as 22.3%. If the assessment criteria were applied exclusively to subjects with a positive result of the MNA-SF screening (following the GLIM diagnostics algorithm), the diagnosis would be overlooked in almost half (41.0%) of persons with malnutrition (Figure 1).

The most frequent phenotypic criterion was weight loss, present in more than one out of four subjects (Table 2). Every fifth participant had low muscle mass (low ALM index). Over 15% of the study population reported reduced food intake, and more than 90% had a disease burden or an inflammatory condition. One-third (30.8%) of the study population had severe malnutrition (stage 1), while the others had moderate malnutrition.

### 3.3. Sensitivity, Specificity, Accuracy, and Diagnostic Value of MNA-SF

Both the sensitivity and specificity of the MNA-SF against the GLIM criteria were fair (59.2% and 78.8%, respectively). The AUC was 0.77, indicating a fair diagnostic value of MNA-SF to diagnose malnutrition. Accuracy was 71.4%, and the agreement between the MNA-SF score and the GLIM criteria was fair (kappa coefficient 0.33). The positive predictive value was 62.9%, while NPV was 76.1%.

## 4. Discussion

The MNA-SF is a simple and cheap tool widely used to assess nutritional status in elderly subjects [27]. It has been validated in numerous countries, including Poland [28]. The diagnostics performance against the GLIM criteria for malnutrition was assessed in the present study.

According to the recognized standards, a tool of high diagnostic value should have sensitivity and specificity higher than 80% and an AUC above 0.8 [26]. The values of the MNA-SF sensitivity, specificity, and the AUC (59.2%, 78.8%, and 0.77, respectively) calculated in the present study indicate a fair diagnostics value of this questionnaire. Based on the GLIM criteria (regardless of the MNA-SF score), 103 (37.7%) participants were malnourished. According to the MNA-SF score, 97 (35.5%) subjects had an impaired nutritional status, 20 (7.3%) had malnutrition, and 77 (28.2%) had a risk of malnutrition. Importantly, only 61 subjects (22.3%) fulfilled the GLIM criteria and had an MNA-SF score to indicate impaired nutritional status. Thus, as many as 42 participants with malnutrition had negative MNA-SF screening results. However, the MNA-SF results were false positive in 36 persons. A possible explanation for this discrepancy may be the different assessment of disease burden in each approach. While the MNA-SF contains a question about acute disease or psychological stress in the past three months, the GLIM etiologic criterion refers to the presence of a chronic disease associated with a permanent or periodic inflammatory status, such as chronic obstructive pulmonary disease or heart failure, even if no exacerbation has recently occurred. Therefore, it is justifiable to introduce clinical suspicion interchangeably with a screening questionnaire in the first step of the diagnostic algorithm for malnutrition. Such an attempt was already used in the diagnosis of sarcopenia [29].

Only two studies investigated the psychometric properties of the MNA-SF against the GLIM criteria in elderly subjects [23,24]. Matsumoto et al. [23] assessed the nutritional status of 490 patients attending an emergency department (mean age of 69.5 ± 16 years); 300 of them (61.2%) were more than 70 years old. Based on the MNA-SF questionnaire, malnutrition or risk of malnutrition was diagnosed in 166 (56.0%) patients aged more than 70 years, while the GLIM criteria were fulfilled in 125 (41.0%) of elderly subjects. In the whole study population, regardless of age, the accuracy of MNA-SF was excellent (97.7%). Those findings contradict the results the results of the present study [23]. Notably, patients in an emergency department are more likely to have an exacerbation of a chronic condition, which may alter the results of the MNA-SF score and result in a better agreement between this screening tool and the GLIM criteria. In the analysis performed by Sobrini et al. [24], the MNA-SF was used as a malnutrition screening tool in 40 oncologic patients aged over 70 years (mean age of 84.8 ± 5.5 years) at risk of frailty. Most subjects (80%; *n* = 32) had malnutrition or risk of malnutrition based on the MNA-SF score. The diagnosis of malnutrition was confirmed with GLIM criteria in 23 subjects (57.5%). Therefore, the sensitivity of MNA-SF was very high (100%), but the GLIM sensitivity was fair (50.0%) [24]. The AUC in the population studied by Sobrini et al. (0.75) was similar to the AUC found in the present study. However, it is difficult to compare the results of both studies, as active cancer was an exclusion criterion in our analysis [24].

Psychometric properties against the GLIM criteria of some other screening tools for malnutrition, such as the Malnutrition Screening Tool (MST), MUST, SGA, and NRS-2002, were also investigated in various populations, including elderly subjects [20,30]. Clark et al. [30] compared the prevalence of malnutrition diagnosed based on the GLIM and ESPEN criteria and MST in a group of 444 geriatric rehabilitation patients (mean age of 82.4 ± 8.01 years). The latter test involves only weight loss and reduction in food intake. The lowest prevalence of malnutrition was found when the ESPEN criteria were applied (12.6%). When etiologic and phenotypic GLIM criteria were used, the prevalence of malnutrition was four times more frequent (52.0%). With the MST screening tool, the risk of malnutrition was diagnosed in 44.4% of patients. Based on its sensitivity and specificity against the GLIM criteria (56.7% and 69.0%, respectively), and an AUC equal to 0.63, the MST diagnostic accuracy appeared fair, similar to our results concerning the MNA-SF [30].

The psychometric properties of the MUST, SGA, and NRS-2002 questionnaires as screening tools in the GLIM algorithm were assessed by Bellanti et al. [20] in a group of 152 elderly patients of the Internal and Aging Medicine Clinic (mean age of 77.8 ± 7.8 years). Based on the GLIM assessment criteria, almost half of the participants had malnutrition (46.0%; *n* = 70). The sensitivity and specificity of the screening tools were as follows: MUST, 64.3% and 81.7%; SGA, 95.7% and 14.6%; NRS-2002, 47.1% and 75.6%. The AUC for the three questionnaires was 0.80, 0.77, and 0.69, respectively. Based on their study, Bellanti et al. concluded the MUST questionnaire is the most suitable screening tool for diagnosing malnutrition in hospitalized elderly subjects [20]. The sensitivity and specificity of the MUST questionnaire are similar to those of the MNA-SF tool in our study.

The limitations of the study are as follows: We utilized information about weight loss and reduced food intake given by the participants in the MNA-SF questionnaire for the phenotypic GLIM criteria. We used the following items: (a) has food intake been declined over the past 3 months due to loss of appetite, digestive problems, chewing, or swallowing difficulties? (answer options: severe decrease in food intake/moderate decrease in food intake/no decrease in food intake); (b) weight loss during the last 3 months? (answer options: weight loss greater than 3 kg/does not know/weight loss between 1 and 3 kg/no weight loss). Many older adults have a problem noticing even short-term changes in body weight, which might have biased their answers.

In terms of study strengths, the present study is one of the first attempts to assess the diagnostic performance of the MNA-SF as a screening tool for malnutrition diagnosis according to the GLIM criteria. It was conducted in a large group of community-dwelling elderly subjects. Previous studies included people of various ages [23] or were limited to elderly patients with cancer [24]. Thus, our results close an important gap in the field.

## 5. Conclusions

The GLIM experts recommend assessing malnutrition phenotypic and etiologic criteria exclusively in subjects with a positive screening test result. However, as Da Silva Passos and De-Souza first noticed in their Letter to the Editor in 2019 [31], the GLIM criteria overlap items of validated screening questionnaires, which biases its sensitivity and specificity. They suggested that the high sensitivity and low specificity of the GLIM diagnostic criteria make them more appropriate as a screening tool than a malnutrition diagnostic, owing to the high risk of a false positive result. In our opinion, the best solution is an optional replacement of a screening tool in the first step of the GLIM algorithm with clinical suspicion of malnutrition. Such an approach was suggested for diagnosing sarcopenia, which is another fundamental problem in advanced age [29]. However, further research is needed to acknowledge our considerations. It should also be emphasized that the number of research studies concerning the diagnostic performance of the MNA-SF as a screening tool for malnutrition is limited, so our results must be viewed with caution.

## Figures and Tables

**Table 1:** The Characteristics of the total study population and of the subgroups fulfilling or not fulfilling the GLIM criteria for malnutrition.

Characteristics	Total *n* = 273	Malnutrition *n* = 103	Normal Nutritional Status *n* = 170	*p*
Age (years)	72.1 ± 7.7	73.8 ± 9.1	71.1 ± 6.6	0.0532
MNA-SF	11.8 ± 2.5	10.3 ± 2.9	12.7 ± 1.7	0.0000
MNA-SF score ≤ 11	97 (35.5)	61 (59.2%)	35 (20.6)	0.0000
AMTS	10.3 ± 0.7	10.3 ± 0.7	10.8 ± 0.2	0.5031
Number of chronic diseases	3.0 ± 1.6	2.9 ± 1.7	3.0 ± 1.6	0.3017
CRP (mg/L)	3.5 ± 4.7	4.0 ± 5.8	3.3 ± 3.9	0.6329
ALM index (kg/m^2^)	7.1 ± 1.3	6.4 ± 1.2	7.5 ± 1.2	0.0000
Height (cm)	162.7 ± 9.2	161.3 ± 9.4	163.6 ± 9.0	0.0396
Weight (kg)	74.2 ± 18.4	63.0 ± 16.6	80.9 ± 16.0	0.0000
BMI (kg/m^2^)	27.9 ± 6.1	24.1 ± 5.6	30.2 ± 5.3	0.0000
BFM (kg)	26.7 ± 11.9	19.9 ± 10.7	30.9 ± 10.7	0.0000
SMM (kg)	25.9 ± 6.3	23.3 ± 5.6	27.5 ± 6.2	0.0000
PBF (%)	34.7 ± 10.1	30.0 ± 10.3	37.6 ± 8.8	0.0000
FFM (kg)	47.5 ± 10.5	43.1 ± 9.3	50.1 ± 10.4	0.0000

Notes: Values are presented as numbers (%) or mean ± standard deviation for descriptive analyses. MNA-SF, Mini Nutritional Assessment-Short Form; AMTS, Abbreviated Mental Test Score; CRP, C Reactive Protein; ALM index, appendicular lean mass index; BMI, body mass index; BFM, body fat mass; SMM, skeletal muscle mass; PBF, percent body fat; FFM, free fat mass.

**Table 2:** Prevalence of the GLIM criteria and impaired nutritional status based on the MNA-SF score.

Criterion	Prevalence, *n* (%) Total *n* = 273
GLIM phenotypic criteria	
Weight loss	77 (28.2)
Low BMI	34 (12.4)
Low ALM index	55 (20.1)
Any phenotypic criteria	104 (38.1)
GLIM etiologic criteria	
Reduce food intake	46 (16.8)
Disease burden or inflammatory condition	256 (93.8)
Any etiologic criteria	268 (98.2)
MNA-SF nutritional status	
Malnutrition	20 (7.3)
Malnutrition risk	77 (28.2)
No malnutrition	176 (64.5)

Note: Values are presented as numbers (%). GLIM, Global Leadership Initiative on Malnutrition; BMI, body mass index; ALM index, appendicular lean mass index; MNA-SF, Mini Nutritional Assessment-Short Form.

## Data Availability

All relevant data are within the manuscript and are openly available in the Zenodo repository (doi:10.5281/zenodo.4837317).
